# Supplementary material for: G-protein coupled estrogen receptor 1, amyloid-β, and tau tangles in older adults
Source: Commun Biol. 2024 May 15;7:569. doi: 10.1038/s42003-024-06272-9 (PMC11096330; doi:10.1038/s42003-024-06272-9)
Supplement: Supplementary file 2 — Supplementary Information [file 42003_2024_6272_MOESM2_ESM.pdf]

Content | **Pages**

Content	Pages
**Supplementary Table 1. Associations of *GPER1* RNA expression level in the dorsolateral prefrontal cortex (DLPFC) with tau tangle density and with modification of the association between Aβ load and tau tangle density, controlled for estrogen-related medications.**	**3**
**Supplementary Table 2. *GPER1* RNA expression levels in 3 brain region regions: dorsolateral prefrontal cortex (DLPFC), posterior cingulate cortex (PCC), anterior caudate (AC).**	**4**
**Supplementary Table 3. Associations of *GPER1* RNA expression level in the posterior cingulate cortex (PCC) and the anterior caudate (AC) with tau tangles and with modification of the association between Aβ load and tau tangles.**	**5**
**Supplementary Table 4. Associations of *GPER1* RNA expression level in the dorsolateral prefrontal cortex (DLPFC), posterior cingulate cortex (PCC), and the anterior caudate (AC) with Braak stages of tau tangle distribution and with modification of the association between Aβ and tau tangle stages of distribution.**	**6**
**Supplementary Table 5. Associations of *GPER1* RNA expression level in the dorsolateral prefrontal cortex (DLPFC), posterior cingulate cortex (PCC), and the anterior caudate (AC) with Aβ load and stages.**	**7**
**Supplementary Table 6. Correlations of RNA expressions of *GPER1* with its downstream signaling pathways' genes.**	**8**
**Supplementary Table 7. Interaction between RNA expressions and protein levels of GPER1 signaling mechanisms and amyloid-β (Aβ).**	**9**
**Supplementary Table 8. Associations of RNA expressions and protein levels of GPER1 signaling mechanisms with tau tangles with and without terms for amyloid-β (Aβ), *GPER1* RNA level, and their interaction in the models.**	**10**
**Supplementary Table 9. Correlations of RNA expressions of *GPER1* with RNA and protein levels of autophagy genes.**	**11**
**Supplementary Table 10. Interaction between RNA expressions and protein levels of autophagy genes and amyloid-β (Aβ).**	**12**

Supplementary Table 11. Associations of RNA expressions and protein levels of autophagy genes with tau tangles with and without terms for amyloid-β (Aβ), *GPER1* RNA level, and their interaction in the models. **13**

Supplementary Table 12. Interaction between Aβ deposits and RNA expressions of 14 G-protein-coupled receptors classified with GPER1 in the same G-protein-receptors category in the association with tau tangles deposits. **14**

Supplementary Table 13. Characteristics of participants with and without snRNA-seq data. **15**

Supplementary Table 14. Correlations between *GPER1* RNA expression levels in astrocytes, excitatory neurons, and endothelial cells. **16**

Supplementary Figure 1. Astrocytic RNA expression levels of 47 inflammatory cytokines. **17**

Supplementary Table 15. Correlations of RNA expressions of astrocytic *GPER1* with astrocytic RNA expressions of cytokines. **18**

Supplementary Table 16. Interaction between RNA expressions of astrocytic cytokine genes and amyloid-β (Aβ). **19**

Supplementary Table 17. Associations of RNA expressions of astrocytic cytokine genes with tau tangles with and without terms for amyloid-β (Aβ), *GPER1* RNA level, and their interaction in the models. **20**

Supplementary Figure 2. Photomicrographs of both amyloid-β and tau tangle staining in AD cases. **21**

Supplementary Table 18. Amyloid-β (Aβ) load, measured by immunohistochemistry, in 8 brain regions. **22**

Supplementary Table 1. Associations of *GPER1* RNA expression level in the dorsolateral prefrontal cortex (DLPFC) with tau tangle density and with modification of the association between Aβ load and tau tangle density, controlled for estrogen-related medications.

Series	Model terms	Model 1	Model 2
		Estimate (SE), p-value	
A	*GPER1* RNA level	0.118 (0.036), 0.001	0.005 (0.066), 0.945
	Aβ load	0.522 (0.029), <0.001	0.532 (0.029), <0.001
	GPER1×Aβ	—	0.065 (0.032), 0.041
B	*GPER1* RNA level	0.118 (0.036), 0.001	0.008 (0.066), 0.901
	Aβ load	0.523 (0.029), <0.001	0.532 (0.029), <0.001
	GPER1×Aβ	—	0.063 (0.032), 0.046
C	*GPER1* RNA level	0.119 (0.036), 0.001	0.006 (0.066), 0.927
	Aβ load	0.523 (0.029), <0.001	0.533 (0.029), <0.001
	GPER1×Aβ	—	0.064 (0.032), 0.041

In three series of linear regressions, association of *GPER1* RNA expression level was examined with tau tangle density (as the outcome). In each series of linear regressions, model 1 included terms for Aβ measure and *GPER1* RNA expression level and a term for an estrogen-related medication. Series A was controlled for estrogens, series B for selective estrogen receptor modulators, and series C for aromatase inhibitors. Model 2 included model 1 terms and a term for an interaction between the Aβ measure and *GPER1* RNA expression level. All the models were also controlled for age at death, sex, and education.

Supplementary Table 2. ***GPER1*** **RNA expression levels in 3 brain region regions: dorsolateral prefrontal cortex (DLPFC), posterior cingulate cortex (PCC), anterior caudate (AC).**

Brain region	Sample size	GPER1 RNA level[a]	Spearman correlation coefficient, p-value	
			PCC	AC
DLPFC	1206	13.8 (0.91)	0.54, <0.001	0.51, <0.001
PCC	633	13.9 (0.67)	—	0.65, <0.001
AC	687	14.3 (0.52)	—	—

[a]Repeated Measure Analysis of Variance indicates that *GPER1* RNA levels in the 3 brain regions were different ($F=85.9$, $p<0.001$). A post hoc Tukey test indicated that all pairwise comparisons were significant at $p<0.05$.

Supplementary Table 3. Associations of *GPER1* RNA expression level in the posterior cingulate cortex (PCC) and the anterior caudate (AC) with tau tangles and with modification of the association between Aβ load and tau tangles.

Series	Model terms	Model 1	Model 2
		Estimate (SE), p-value	
A	*GPER1* RNA level in PCC	0.166 (0.067), 0.014	-0.024 (0.116), 0.837
	Aβ load	0.503 (0.040), <0.001	0.514 (0.040), <0.001
	GPER1_PCC×Aβ load	—	0.121 (0.060), 0.045
B	*GPER1* RNA level in AC	0.079 (0.080), 0.324	0.113 (0.131), 0.389
	Aβ load	0.519 (0.036), <0.001	0.526 (0.041), <0.001
	GPER1_AC×Aβ load	—	-0.022 (0.067), 0.741

In two series of linear regressions, associations of *GPER1* RNA expression levels at PCC and AC were examined with tau tangles density (as the outcome). In each series of linear regressions, *GPER1* RNA expression level in either PCC (Series A) or AC (series B) was examined in two models. Model 1 included terms for Aβ measure and *GPER1* RNA expression level, and model 2 included model 1 terms and a term for an interaction between the Aβ measure and *GPER1* RNA expression level. All the models were controlled for age at death, sex, and education.

Supplementary Table 4. Associations of *GPER1* RNA expression level in the dorsolateral prefrontal cortex (DLPFC), posterior cingulate cortex (PCC), and the anterior caudate (AC) with Braak stages of tau tangle distribution and with modification of the association between Aβ and tau tangle stages of distribution.

Series	Model terms	Model 1	Model 2
		**OR (95%CI), p-value**	**Estimate (SE), p-value**
A	*GPER1* RNA level in DLPFC	1.13 (1.00–1.27), 0.042	-0.080 (0.128), 0.533
	Aβ stage	3.10 (2.76–3.49), <0.001	1.15 (0.060), <0.001
	GPER1_DLPFC×Aβ stage	—	0.106 (0.058), 0.069
B	*GPER1* RNA level in PCC	1.15 (0.92–1.43), 0.223	-0.217 (0.229), 0.345
	Aβ stage	3.17 (2.70–3.73), <0.001	1.18 (0.084), <0.001
	GPER1_PCC×Aβ stage	—	0.182 (0.103), 0.077
C	*GPER1* RNA level in AC	1.16 (0.88–1.52), 0.302	0.085 (0.261), 0.744
	Aβ stage	3.06 (2.63–3.57), <0.001	1.11 (0.086), <0.001
	GPER1_AC×Aβ stage	—	0.033 (0.123), 0.788

In three series of ordinal logistic regressions, associations of *GPER1* RNA expression levels at 3 brain regions (DLPFC, PCC, and AC) were examined with Braak tau tangle stages (as the outcome). In each series of logistic regressions, *GPER1* RNA expression level in either DLPFC (series A), PCC (Series B) or AC (series C) was examined in two models. Model 1 included terms for modified Thal Aβ stages and *GPER1* RNA expression level, and model 2 included model 1 terms and a term for an interaction between the Aβ stage and *GPER1* RNA expression level. All the models were controlled for age at death, sex, and education. Please note that because of logistic regressions and using a 4-level Braak stages as the outcome, Model 1 cells include odds ratios (OR) and their 95% confidence intervals. However, Model 2 cells include estimates and standard errors as model 2 has the interaction terms.

Supplementary Table 5. Associations of *GPER1* RNA expression level in the dorsolateral prefrontal cortex (DLPFC), posterior cingulate cortex (PCC), and the anterior caudate (AC) with Aβ load and stages.

**Model terms**	**Aβ load**	**Aβ stages**
	**Estimate (SE), p-value**	**OR (95%CI), p-value**
GPER1 RNA level in DLPFC	-0.032 (0.032), 0.317	1.00 (0.88–1.13), 0.964
GPER1 RNA level in PCC	0.041 (0.060), 0.492	1.17 (0.93–1.46), 0.178
GPER1 RNA level in AC	0.060 (0.075), 0.421	1.17 (0.89–1.55), 0.258

In separate linear and ordinal logistic regressions, associations of *GPER1* RNA expression levels at 3 brain regions (DLPFC, PCC, and AC) were examined with Aβ load (linear regressions) and Aβ stages (ordinal logistic regressions) as the outcome. The linear regression models were controlled for tau tangle density and the logistic regressions for tau tangle stages. In addition, all the models were controlled for age at death, sex, and education.

Supplementary Table 6. Correlations of RNA expressions of *GPER1* with its downstream signaling pathways' genes.

Genes	Ensemble	Uniprot. ID	Spearman Correlation coefficient, p-value	
			RNA	Protein
JNK1	ENSG00000107643	P45983	-0.28, <0.001	-0.22, <0.001
JNK2	ENSG00000050748	P45984	-0.24, <0.001	-0.23, <0.001
JNK3	ENSG00000109339	P53779	-0.20, <0.001	-0.21, <0.001
ERK1	ENSG00000102882	P27361	0.01, 0.845	0.11, 0.003
ERK2	ENSG00000100030	P28482	-0.02, 0.477	0.11, 0.002
Akt1	ENSG00000142208	P31749	0.14, <0.001	-0.1, 0.006
Akt2	ENSG00000105221	P31751	0.06, 0.040	-0.05, 0.213
Akt3	ENSG00000117020	Q9Y243	-0.17, <0.001	-0.05, 0.219
ADCY1	ENSG00000164742	Q08828	-0.22, <0.001	-0.12, 0.002
ADCY2	ENSG00000078295	Q08462	0.01, 0.650	-0.27, <0.001
ADCY3	ENSG00000138031	O60266	-0.03, 0.244	-0.14, <0.001
ADCY4	ENSG00000129467	Q8NFM4	0.03, 0.237	NA
ADCY5	ENSG00000173175	O95622	-0.01, 0.785	-0.09, 0.18
ADCY6	ENSG00000174233	O43306	-0.01, 0.759	-0.07, 0.068
ADCY7	ENSG00000121281	P51828	0.10, <0.001	-0.19, <0.001
ADCY8	ENSG00000155897	P40145	0.03, 0.304	-0.07, 0.058
ADCY9	ENSG00000162104	O60503	0.18, <0.001	0.04, 0.225
ADCY10	ENSG00000143199	Q96PN6	-0.04, 0.187	NA
PRKACA	ENSG00000072062	P17612	-0.24, <0.001	0.02, 0.66
PRKAR2B	ENSG00000005249	P31323	-0.14, <0.001	-0.02, 0.649
PRKAR1B	ENSG00000188191	P31321	-0.20, <0.001	-0.04, 0.279
PRKACB	ENSG00000142875	P22694	-0.23, <0.001	0.13, <0.001
PRKAR2A	ENSG00000114302	P13861	0.06, 0.028	0, 0.953
PRKAR1A	ENSG00000108946	P10644	-0.19, <0.001	0.01, 0.72
PLCB1	ENSG00000182621	Q9NQ66	-0.19, <0.001	-0.2, <0.001
PLCB2	ENSG00000137841	Q00722	0.04, 0.208	-0.19, <0.001
PLCB3	ENSG00000149782	Q01970	0.35, <0.001	-0.07, 0.042
PLCB4	ENSG00000101333	Q15147	-0.02, 0.426	0.01, 0.711

NA: Protein levels of two genes were not quantified after implementing quality control.

Supplementary Table 7. Interaction between RNA expressions and protein levels of GPER1 signaling mechanisms and amyloid-β (Aβ).

Genes	Ensemble	Uniprot. ID	Interaction with Aβ Estimate (SE), q-value	
			RNA	Protein
JNK1	**ENSG00000107643**	**P45983**	-0.309 (0.120), 0.096	-0.774 (0.48), 0.254
JNK2	**ENSG00000050748**	**P45984**	-0.141 (0.088), 0.247	-0.897 (0.493), 0.200
JNK3	**ENSG00000109339**	**P53779**	-0.062 (0.095), 0.628	-0.553 (0.523), 0.526
ERK1	**ENSG00000102882**	**P27361**	-0.057 (0.155), 0.800	0.453 (0.240), 0.200
ERK2	**ENSG00000100030**	**P28482**	0.001 (0.208), 0.998	0.716 (0.270), 0.116
Akt1	**ENSG00000142208**	**P31749**	-0.068 (0.182), 0.800	-0.561 (0.568), 0.526
Akt2	**ENSG00000105221**	**P31751**	-0.097 (0.149), 0.628	-1.08 (0.510), 0.179
Akt3	**ENSG00000117020**	**Q9Y243**	-0.213 (0.181), 0.435	-1.248 (0.496), 0.116
ADCY1	**ENSG00000164742**	**Q08828**	-0.214 (0.090), 0.099	-0.769 (0.345), 0.17
ADCY2	**ENSG00000078295**	**Q08462**	0.018 (0.095), 0.879	-0.487 (0.282), 0.220
ADCY3	**ENSG00000138031**	**O60266**	0.144 (0.140), 0.450	-0.107 (0.252), 0.872
ADCY4	**ENSG00000129467**	**Q8NFM4**	-0.054 (0.051), 0.450	NA
ADCY5	**ENSG00000173175**	**O95622**	0.214 (0.135), 0.247	0.11 (0.384), 0.915
ADCY6	**ENSG00000174233**	**O43306**	-0.238 (0.138), 0.247	-0.076 (0.231), 0.915
ADCY7	**ENSG00000121281**	**P51828**	0.100 (0.064), 0.247	-0.401 (0.286), 0.348
ADCY8	**ENSG00000155897**	**P40145**	0.078 (0.084), 0.490	0.611 (0.335), 0.200
ADCY9	**ENSG00000162104**	**O60503**	0.148 (0.109), 0.345	-0.194 (0.374), 0.828
ADCY10	**ENSG00000143199**	**Q96PN6**	0.068 (0.037), 0.242	NA
PRKACA	**ENSG00000072062**	**P17612**	-0.284 (0.118), 0.099	-0.19 (0.238), 0.651
PRKAR2B	**ENSG00000005249**	**P31323**	-0.028 (0.094), 0.827	0.048 (0.283), 0.961
PRKAR1B	**ENSG00000188191**	**P31321**	-0.182 (0.087), 0.166	-0.227 (0.408), 0.828
PRKACB	**ENSG00000142875**	**P22694**	-0.150 (0.135), 0.436	-0.009 (0.186), 0.961
PRKAR2A	**ENSG00000114302**	**P13861**	0.438 (0.163), 0.096	-0.047 (0.361), 0.961
PRKAR1A	**ENSG00000108946**	**P10644**	-0.312 (0.168), 0.242	0.353 (0.357), 0.526
PLCB1	**ENSG00000182621**	**Q9NQ66**	**-0.385 (0.084), <0.001**	-0.605 (0.244), 0.116
PLCB2	**ENSG00000137841**	**Q00722**	0.062 (0.054), 0.435	-0.458 (0.230), 0.200
PLCB3	**ENSG00000149782**	**Q01970**	0.128 (0.080), 0.247	-0.028 (0.325), 0.961
PLCB4	**ENSG00000101333**	**Q15147**	-0.057 (0.084), 0.628	0.398 (0.386), 0.526

In 28 linear regressions, we separately examined interactions between RNA expressions of GPER1downstream signaling mechanisms and Aβ load in the associations with tau tangles deposits. Each model included RNA expression of one of the genes listed in the left column, Aβ, and their interaction. All models were controlled for age at death, sex, and education. Next, in a separate series of 26 linear regressions we replaced RNA expression levels with protein levels of GPER1downstream signaling mechanisms and repeated the analysis.

Supplementary Table 8. Associations of RNA expressions and protein levels of GPER1 signaling mechanisms with tau tangles with and without terms for amyloid-β (Aβ), *GPER1* RNA level, and their interaction in the models.

Genes	RNA		Protein	
	**Model A**	**Model B**	**Model A**	**Model B**
JNK1	**-0.540 (0.158), 0.003**	-0.148 (0.144), 0.465	-1.159 (0.653), 0.142	-0.556 (0.579), 0.548
JNK2	-0.090 (0.116), 0.511	0.064 (0.105), 0.628	-1.272 (0.663), 0.111	-0.64 (0.588), 0.548
JNK3	-0.030 (0.122), 0.867	0.109 (0.109), 0.465	-0.897 (0.697), 0.323	-0.242 (0.616), 0.784
ERK1	**0.492 (0.201), 0.045**	0.247 (0.178), 0.309	**1.511 (0.313), <0.001**	**0.687 (0.282), 0.043**
ERK2	**0.720 (0.258), 0.019**	0.500 (0.228), 0.132	**2.071 (0.345), <0.001**	**1.237 (0.31), 0.001**
Akt1	-0.005 (0.242), 0.996	-0.118 (0.213), 0.651	-0.407 (0.783), 0.768	-0.216 (0.686), 0.799
Akt2	-0.374 (0.194), 0.090	-0.327 (0.171), 0.154	-1.573 (0.71), 0.059	-1.054 (0.621), 0.213
Akt3	**-0.560 (0.239), 0.045**	-0.237 (0.212), 0.434	**-1.921 (0.655), 0.008**	**-1.378 (0.573), 0.043**
ADCY1	-0.252 (0.124), 0.073	-0.07 (0.111), 0.628	**-2.612 (0.436), <0.001**	**-1.39 (0.395), 0.003**
ADCY2	-0.118 (0.118), 0.428	-0.052 (0.104), 0.662	**-1.092 (0.373), 0.008**	-0.482 (0.336), 0.33
ADCY3	0.145 (0.179), 0.511	0.052 (0.158), 0.744	0.209 (0.348), 0.751	0.309 (0.306), 0.548
ADCY4	**-0.245 (0.065), 0.003**	**-0.237 (0.057), 0.001**	NA	NA
ADCY5	**0.625 (0.174), 0.003**	0.439 (0.154), 0.061	-0.183 (0.514), 0.852	0.435 (0.451), 0.548
ADCY6	**-0.432 (0.18), 0.045**	-0.312 (0.159), 0.154	0.147 (0.297), 0.768	0.213 (0.26), 0.623
ADCY7	-0.064 (0.081), 0.511	-0.057 (0.072), 0.566	**-1.804 (0.378), <0.001**	**-0.893 (0.341), 0.029**
ADCY8	0.244 (0.109), 0.052	0.181 (0.096), 0.154	**1.784 (0.466), <0.001**	**1.472 (0.409), 0.003**
ADCY9	**0.495 (0.139), 0.003**	0.259 (0.125), 0.154	-0.304 (0.502), 0.751	-0.312 (0.437), 0.644
ADCY10	0.06 (0.049), 0.313	0.075 (0.043), 0.183	NA	NA
PRKACA	**-0.489 (0.144), 0.003**	-0.200 (0.131), 0.253	-0.095 (0.392), 0.877	-0.269 (0.342), 0.623
PRKAR2B	0.222 (0.119), 0.097	0.237 (0.105), 0.132	-0.012 (0.374), 0.974	-0.083 (0.326), 0.799
PRKAR1B	-0.145 (0.117), 0.313	-0.049 (0.105), 0.662	0.386 (0.574), 0.751	0.341 (0.5), 0.644
PRKACB	-0.001 (0.168), 0.996	0.130 (0.151), 0.545	0.521 (0.334), 0.206	0.115 (0.293), 0.784
PRKAR2A	0.44 (0.206), 0.061	0.363 (0.181), 0.154	-0.031 (0.485), 0.974	-0.246 (0.423), 0.695
PRKAR1A	**-0.529 (0.224), 0.045**	-0.236 (0.201), 0.424	**1.518 (0.473), 0.004**	**1.102 (0.414), 0.029**
PLCB1	**-0.438 (0.108), 0.002**	-0.236 (0.098), 0.132	**-1.885 (0.312), <0.001**	**-1.114 (0.282), 0.001**
PLCB2	-0.021 (0.069), 0.857	-0.046 (0.061), 0.579	**-1.711 (0.292), <0.001**	**-0.898 (0.265), 0.004**
PLCB3	**0.356 (0.101), 0.003**	0.161 (0.093), 0.183	0.108 (0.448), 0.877	-0.108 (0.393), 0.799
PLCB4	0.241 (0.107), 0.052	0.216 (0.094), 0.132	**2.011 (0.495), <0.001**	**1.207 (0.437), 0.026**

The cell's numbers are estimates (SE), q-values, illustrating associations of RNA expressions (2nd and 3rd columns) and protein levels (4th and 5th columns) of the genes of GPER1 signaling mechanisms with tau tangles as the outcome. Model A terms are RNA expression or protein levels of one of the genes listed in the left column. Model B terms include corresponding Model A term, *GPER1* RNA expression level, Aβ, and interaction between *GPER1* and Aβ. Both Models A and B were controlled for age at death, sex, and education.

Supplementary Table 9. Correlations of RNA expressions of *GPER1* with RNA and protein levels of autophagy genes.

Genes	Ensemble	Uniprot. ID	Spearman Correlation coefficient, p-value	
			RNA	Protein
ULK1	**ENSG00000177169**	**O75385**	-0.03, 0.369	-0.04, 0.23
ATG14	**ENSG00000126775**	**Q6ZNE5**	**-0.19, <0.001**	0.02, 0.597
BCL2	**ENSG00000171791**	**P10415**	**0.19, <0.001**	0.01, 0.85
BECN1	**ENSG00000126581**	**Q14457**	**-0.08, 0.004**	**-0.11, 0.004**
PIK3C3	**ENSG00000078142**	**Q8NEB9**	**-0.21, <0.001**	**-0.12, 0.001**
UVRAG	**ENSG00000198382**	**Q9P2Y5**	**-0.06, 0.03**	**-0.08, 0.038**
ATG4A	**ENSG00000101844**	**NA**	**0.09, 0.002**	NA
ATG4B	**ENSG00000168397**	**Q9Y4P1**	0.02, 0.468	**0.13, <0.001**
ATG4C	**ENSG00000125703**	**Q96DT6**	**0.24, <0.001**	0.06, 0.136
ATG4D	**ENSG00000130734**	**Q86TL0**	**0.08, 0.005**	0.03, 0.604
ATG7	**ENSG00000197548**	**O95352**	**-0.2, <0.001**	-0.03, 0.352
ATG10	**ENSG00000152348**	**NA**	**-0.06, 0.038**	NA
ATG12	**ENSG00000145782**	**O94817**	0.01, 0.804	0.03, 0.392
BNIP3	**ENSG00000176171**	**Q12983**	**-0.09, 0.002**	**-0.21, <0.001**
MAP1LC3A	**ENSG00000101460**	**Q9H492**	**-0.27, <0.001**	**-0.17, <0.001**
MAP1LC3B	**ENSG00000140941**	**NA**	**-0.13, <0.001**	NA
SQSTM1	**ENSG00000161011**	**Q13501**	**0.06, 0.043**	0, 0.984
ATG2A	**ENSG00000110046**	**Q2TAZ0**	**-0.2, <0.001**	**-0.1, 0.007**
ATG2B	**ENSG00000066739**	**Q96BY7**	**-0.21, <0.001**	-0.04, 0.303
ATG9A	**ENSG00000198925**	**Q7Z3C6**	**-0.09, 0.001**	**0.16, <0.001**
ATG9B	**ENSG00000181652**	**NA**	**-0.22, <0.001**	NA
WIPI2	**ENSG00000157954**	**Q9Y4P8**	0.02, 0.406	0.04, 0.341

NA: Protein levels of four genes were not quantified after implementing quality control.

Supplementary Table 10. Interaction between RNA expressions and protein levels of autophagy genes and amyloid-β (Aβ).

Genes	Ensemble	Uniprot. ID	Interaction with Aβ Estimate (SE), q-value	
			RNA	Protein
ULK1	**ENSG00000177169**	**O75385**	0.108 (0.095), 0.379	-0.398 (0.317), 0.326
ATG14	**ENSG00000126775**	**Q6ZNE5**	-0.241 (0.111), 0.11	-0.001 (0.337), 0.997
BCL2	**ENSG00000171791**	**P10415**	0.216 (0.088), 0.107	0.423 (0.221), 0.170
BECN1	**ENSG00000126581**	**Q14457**	0.048 (0.130), 0.743	-0.728 (0.524), 0.326
PIK3C3	**ENSG00000078142**	**Q8NEB9**	-0.527 (0.175), 0.057	-0.73 (0.591), 0.326
UVRAG	**ENSG00000198382**	**Q9P2Y5**	0.236 (0.156), 0.241	-0.036 (0.555), 0.997
ATG4A	**ENSG00000101844**	**NA**	0.138 (0.128), 0.386	NA
ATG4B	**ENSG00000168397**	**Q9Y4P1**	0.061 (0.133), 0.714	0.436 (0.43), 0.431
ATG4C	**ENSG00000125703**	**Q96DT6**	0.183 (0.080), 0.108	0.243 (0.141), 0.193
ATG4D	**ENSG00000130734**	**Q86TL0**	0.195 (0.123), 0.224	-0.099 (0.234), 0.866
ATG7	**ENSG00000197548**	**O95352**	-0.419 (0.170), 0.107	-0.069 (0.453), 0.990
ATG10	**ENSG00000152348**	**NA**	-0.126 (0.079), 0.224	NA
ATG12	**ENSG00000145782**	**O94817**	-0.197 (0.170), 0.379	-0.33 (0.266), 0.326
BNIP3	**ENSG00000176171**	**Q12983**	-0.279 (0.124), 0.108	-0.517 (0.195), 0.050
MAP1LC3A	**ENSG00000101460**	**Q9H492**	-0.22 (0.105), 0.112	**-1.58 (0.397), 0.001**
MAP1LC3B	**ENSG00000140941**	**NA**	-0.196 (0.143), 0.290	NA
SQSTM1	**ENSG00000161011**	**Q13501**	0.036 (0.109), 0.743	**0.623 (0.192), 0.011**
ATG2A	**ENSG00000110046**	**Q2TAZ0**	-0.114 (0.139), 0.503	-0.984 (0.508), 0.170
ATG2B	**ENSG00000066739**	**Q96BY7**	-0.322 (0.170), 0.160	-1.100 (0.639), 0.193
ATG9A	**ENSG00000198925**	**Q7Z3C6**	0.173 (0.179), 0.430	0.703 (0.278), 0.053
ATG9B	**ENSG00000181652**	**NA**	-0.083 (0.052), 0.224	NA
WIPI2	**ENSG00000157954**	**Q9Y4P8**	0.150 (0.205), 0.538	-0.070 (0.407), 0.990

In 22 linear regressions, we separately examined interactions between RNA expressions of autophagy genes and Aβ load in the associations with tau tangles. Each model included RNA expression of one of the genes listed in the left column, Aβ, and their interaction. All models were controlled for age at death, sex, and education. Next, in a separate series of 18 linear regressions we replaced RNA expression levels with protein levels of autophagy genes and repeated the analysis.

Supplementary Table 11. Associations of RNA expressions and protein levels of autophagy genes with tau tangles with and without terms for amyloid-β (Aβ), *GPER1* RNA level, and their interaction in the models.

Genes	RNA		Protein	
	**Model A**	**Model B**	**Model A**	**Model B**
ULK1	0.179 (0.127), 0.216	0.171 (0.111), 0.249	-0.583 (0.417), 0.252	-0.244 (0.365), 0.732
ATG14	**-0.588 (0.142), <0.001**	**-0.389 (0.128), 0.030**	0.450 (0.483), 0.422	0.179 (0.422), 0.806
BCL2	**0.312 (0.114), 0.015**	0.146 (0.102), 0.279	**1.116 (0.287), <0.001**	**0.823 (0.257), 0.007**
BECN1	-0.107 (0.174), 0.567	0.059 (0.154), 0.774	-1.055 (0.680), 0.219	-0.372 (0.599), 0.732
PIK3C3	**-0.972 (0.221), <0.001**	**-0.572 (0.199), 0.030**	-1.814 (0.812), 0.058	-0.714 (0.717), 0.678
UVRAG	0.413 (0.193), 0.059	0.296 (0.170), 0.212	-0.784 (0.720), 0.356	-0.118 (0.630), 0.943
ATG4A	0.114 (0.158), 0.516	-0.006 (0.139), 0.968	NA	NA
ATG4B	**0.474 (0.169), 0.014**	0.307 (0.150), 0.137	0.780 (0.589), 0.258	0.422 (0.516), 0.678
ATG4C	**0.354 (0.104), 0.002**	0.086 (0.095), 0.499	0.311 (0.175), 0.153	0.208 (0.153), 0.449
ATG4D	**0.61 (0.156), <0.001**	**0.405 (0.139), 0.030**	-0.039 (0.295), 0.895	-0.147 (0.259), 0.732
ATG7	**-0.638 (0.213), 0.009**	-0.222 (0.192), 0.386	0.499 (0.620), 0.474	0.075 (0.543), 0.943
ATG10	-0.129 (0.102), 0.254	-0.140 (0.090), 0.249	NA	NA
ATG12	-0.406 (0.216), 0.096	-0.327 (0.191), 0.212	**-0.892 (0.375), 0.045**	-0.305 (0.331), 0.678
BNIP3	**-0.684 (0.16), <0.001**	-0.374 (0.143), 0.051	**-1.524 (0.268), <0.001**	**-0.936 (0.243), 0.001**
MAP1LC3A	**-0.557 (0.138), <0.001**	-0.253 (0.125), 0.137	**-3.359 (0.531), <0.001**	**-2.125 (0.479), <0.001**
MAP1LC3B	-0.355 (0.189), 0.096	-0.089 (0.168), 0.692	NA	NA
SQSTM1	**-0.342 (0.149), 0.044**	-0.301 (0.132), 0.100	**2.207 (0.258), <0.001**	**1.633 (0.232), <0.001**
ATG2A	**-0.458 (0.172), 0.017**	-0.184 (0.155), 0.386	**-2.484 (0.704), 0.001**	-1.272 (0.624), 0.126
ATG2B	-0.338 (0.210), 0.158	-0.122 (0.190), 0.637	-1.205 (0.873), 0.252	-0.632 (0.763), 0.678
ATG9A	0.053 (0.230), 0.818	0.195 (0.203), 0.495	**1.809 (0.395), <0.001**	0.837 (0.354), 0.066
ATG9B	-0.084 (0.066), 0.254	-0.007 (0.059), 0.945	NA	NA
WIPI2	0.216 (0.260), 0.470	0.170 (0.229), 0.592	0.339 (0.611), 0.613	0.005 (0.533), 0.993

The cell's numbers are estimates (SE), q-values, illustrating associations of RNA expressions (2nd and 3rd columns) and protein levels (4th and 5th columns) of the autophagy genes with tau tangles as the outcome. Model A terms are RNA expression or protein levels of one of the genes listed in the left column. Model B terms include corresponding Model A term, *GPER1* RNA expression level, Aβ, and interaction between *GPER1* and Aβ. Both Models A and B were controlled for age at death, sex, and education.

Supplementary Table 12. Interaction between Aβ deposits and RNA expressions of 14 G-protein-coupled receptors classified with GPER1 in the same G-protein-receptors category in the association with tau tangles deposits.

Genes	**Interaction with Aβ38**
	**Estimate (SE), q-value**
Histamine Receptor H3	-0.029 (0.049), 0.858
G Protein-Coupled Receptor 63	0.009 (0.062), 0.926
G Protein-Coupled Receptor 75	0.067 (0.031), 0.232
Adhesion G Protein-Coupled Receptor V1	-0.057 (0.036), 0.494
G Protein-Coupled Receptor 85	-0.118 (0.08), 0.494
G Protein-Coupled Receptor 176	0.008 (0.037), 0.926
G Protein-Coupled Receptor Class C Group 5 Member B	0.036 (0.073), 0.875
G Protein-Coupled Receptor Class C Group 5 Member C	0.042 (0.07), 0.858
G Protein-Coupled Receptor 34	0.013 (0.09), 0.926
G Protein-Coupled Receptor 22	-0.053 (0.064), 0.814
Vomeronasal 1 Receptor 1	0.069 (0.074), 0.814
G Protein-Coupled Receptor 19	-0.004 (0.047), 0.926
G Protein-Coupled Receptor 39	0.061 (0.062), 0.814
Histamine Receptor H1	-0.143 (0.059), 0.228

In 14 linear regressions, we separately examined interactions between RNA expressions of 14 G-protein-coupled receptors and Aβ deposits in the associations with tau tangles deposits. Each model included RNA expression of one of the genes listed in the left column, Aβ, and their interaction. All models were controlled for age at death, sex, and education.

Supplementary Table 13. Characteristics of participants with and without snRNA-seq data.

Characteristics	Mean (SD) or n (%)		P-value
	With snRNA-seq (n=419)	Without snRNA-seq (n=787)	
Age at death baseline, years, Mean (SD)	89.3 (6.8)	89.7 (6.4)	0.260
Women, n (%)	285 (68.0)	535 (68.0)	0.989
Education, years, Mean (SD)	16.3 (3.5)	16.2 (3.6)	0.576
White non-Hispanic, n (%)	418 (99.8)	749 (95.2)	<0.001
Mini Mental State Examination score, Mean (SD)	21.4 (8.7)	20.6 (9.4)	0.152
Alzheimer's dementia, n (%)	156 (37.2)	351 (44.7)	0.013
Pathological diagnosis of Alzheimer's disease, n (%)	270 (64.8)	505 (64.3)	0.886
Immunohistochemical assessments			
Square root of Amyloid-β deposit levels, Mean (SD)	1.6 (1.2)	1.6 (1.1)	0.430
Square root of Tau tangles deposit levels, Mean (SD)	1.5 (1.2)	1.7 (1.4)	0.011

Supplementary Table 14. Correlations between *GPER1* RNA expression levels in astrocytes, excitatory neurons, and endothelial cells.

**Cells**	**Astrocytes**	**Excitatory neurons**	**Endothelial cells**
**Astrocytes**	1	-0.25, <0.001	0.04, 0.462
**Excitatory neurons**	NA	1	0.01, 0.775
**Endothelial cells**	NA	NA	1

Cells' numbers are Spearman correlation coefficients, p-values.

Supplementary Figure 1. **Astrocytic RNA expression levels of 47 inflammatory cytokines.**

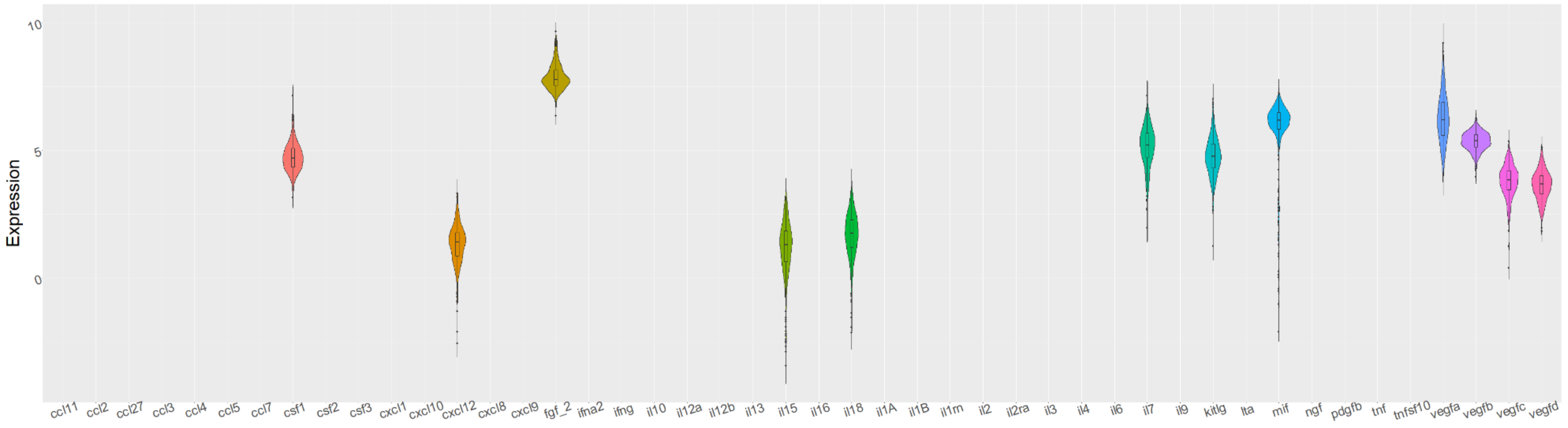

Supplementary Table 15. Correlations of RNA expressions of astrocytic *GPER1* with astrocytic RNA expressions of cytokines.

Genes	**Ensemble**	**Spearman Correlation coefficient, p-value**
csf1	ENSG00000184371	**0.21, <0.001**
cxcl12	ENSG00000107562	0.002, 0.633
fgf	ENSG00000138685	**-0.17, <0.001**
il15	ENSG00000164136	-0.09, 0.66
il18	ENSG00000150782	**0.16, <0.001**
il7	ENSG00000104432	-0.04, 0.432
kitlg	ENSG00000049130	-0.01, 0.817
mif	ENSG00000240972	0.08, 0.097
vegfa	ENSG00000112715	0.06, 0.215
vegfb	ENSG00000173511	**0.19, <0.001**
vegfc	ENSG00000150630	**-0.10, 0.037**
vegfd	ENSG00000165197	**0.12, 0.014**

Supplementary Table 16. Interaction between RNA expressions of astrocytic cytokine genes and amyloid-β (Aβ).

Genes	**Estimate (SE), q-value**
csf1	0.213 (0.075), 0.056
cxcl12	0.066 (0.055), 0.455
fgf	0.045 (0.079), 0.715
il15	0.021 (0.039), 0.715
il18	-0.076 (0.046), 0.371
il7	-0.113 (0.049), 0.128
kitlg	0.041 (0.056), 0.715
mif	0.049 (0.032), 0.371
vegfa	-0.002 (0.046), 0.957
vegfb	0.141 (0.117), 0.455
vegfc	-0.034 (0.061), 0.715
vegfd	0.027 (0.070), 0.767

In 12 linear regressions, we separately examined interactions between RNA expressions of astrocytic cytokine genes and Aβ load in the associations with tau tangles. Each model included RNA expression of one of the genes listed in the left column, Aβ, and their interaction. All models were controlled for age at death, sex, and education.

Supplementary Table 17. Associations of RNA expressions of astrocytic cytokine genes with tau tangles with and without terms for amyloid-β (Aβ), *GPER1* RNA level, and their interaction in the models.

Genes	Model A	Model B
csf1	**0.288 (0.101), 0.030**	0.084 (0.091), 0.796
cxcl12	0.032 (0.073), 0.792	-0.037 (0.064), 0.796
fgf	-0.038 (0.112), 0.800	-0.058 (0.099), 0.796
il15	-0.048 (0.052), 0.558	-0.008 (0.046), 0.894
il18	**-0.158 (0.062), 0.033**	-0.096 (0.056), 0.373
il7	**-0.186 (0.069), 0.030**	-0.135 (0.06), 0.306
kitlg	**0.206 (0.073), 0.030**	0.109 (0.065), 0.373
mif	0.025 (0.039), 0.705	0.010 (0.034), 0.894
vegfa	0.008 (0.058), 0.894	0.007 (0.051), 0.894
vegfb	0.139 (0.156), 0.558	0.073 (0.138), 0.796
vegfc	0.166 (0.085), 0.113	0.055 (0.076), 0.796
vegfd	0.187 (0.098), 0.113	0.049 (0.087), 0.796

The cell's numbers are estimates (SE), q-values, illustrating associations of RNA expressions of astrocytic cytokine genes with tau tangles as the outcome. Model A terms are RNA expression of one of the genes listed in the left column. Model B terms include corresponding Model A term, *GPER1* RNA expression level, Aβ, and interaction between *GPER1* and Aβ. Both Models A and B were controlled for age at death, sex, and education.

Supplementary Figure 2. Photomicrographs of both amyloid-β and tau tangle staining in AD cases.

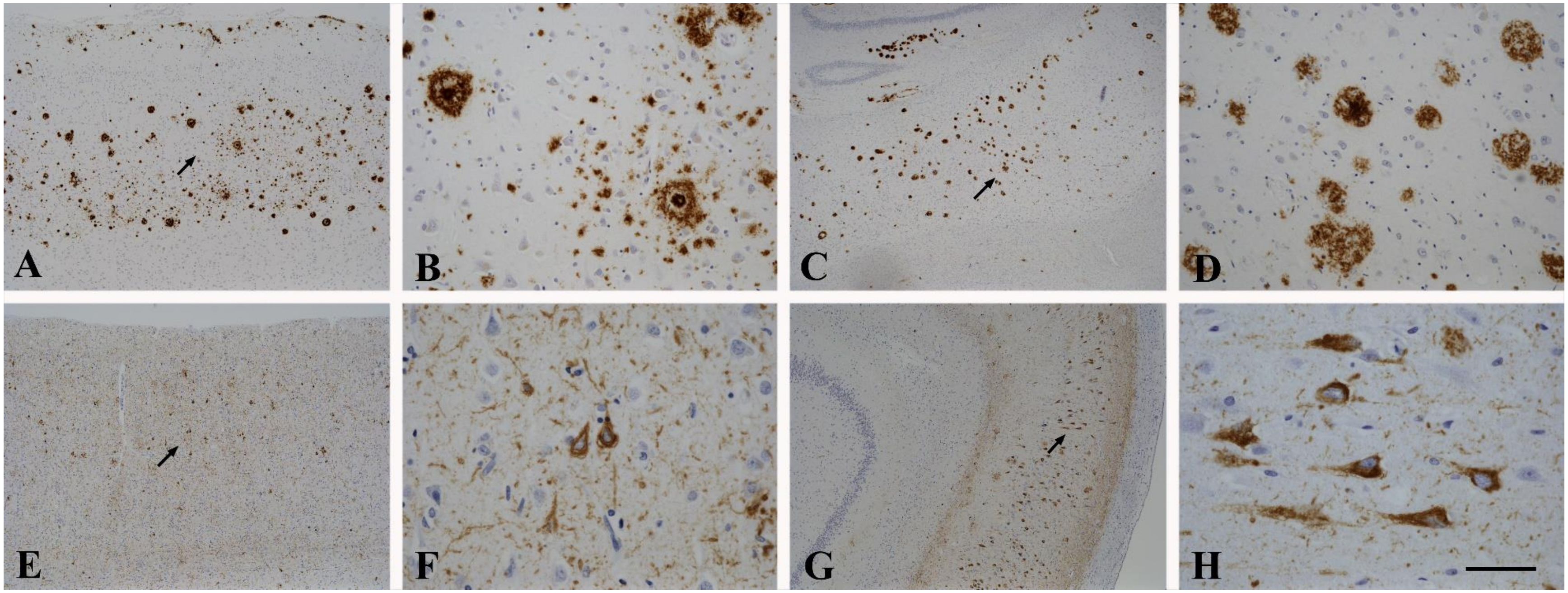

A. Lower magnification photomicrograph of amyloid-β staining from midfrontal cortex. B. Higher magnification of the area indicated by the arrow in A. C. Lower magnification photomicrograph of amyloid-β staining from hippocampus. D. Higher magnification of the area indicated by the arrow in C. E. Lower magnification photomicrograph of tau tangle staining from midfrontal cortex. F. Higher magnification of the area indicated by the arrow in E. G. Lower magnification photomicrograph of tau tangle staining from hippocampus. H. Higher magnification of the area indicated by the arrow in G. Scale bar in H represents the following magnifications: A, E, and G=500 µm, C=1000 µm, B and D=100 µm, F and H=50 µm.

Supplementary Table 18. Amyloid-β (Aβ) load, measured by immunohistochemistry, in 8 brain regions.

Brain region	Square root of Aβ load,[a] mean (SD)	Spearman correlation coefficient, p-value						
		Superior frontal cortex	Hippocamp	Inferior temporal cortex	Entorhinal cortex	Anterior cingulate cortex	Angular gyrus	Calcarine cortex
**Middle frontal cortex**	**1.94 (1.34)**	**0.94, <0.001**	**0.81, <0.001**	**0.91, <0.001**	**0.88, <0.001**	**0.92, <0.001**	**0.93, <0.001**	**0.83, <0.001**
**Superior frontal cortex**	**1.77 (1.31)**	—	**0.86, <0.001**	**0.92, <0.001**	**0.90, <0.001**	**0.95, <0.001**	**0.93, <0.001**	**0.86, <0.001**
**Hippocampus**	**0.94 (0.88)**	—	—	**0.87, <0.001**	**0.89, <0.001**	**0.85, <0.001**	**0.84, <0.001**	**0.84, <0.001**
**Inferior temporal cortex**	**1.70 (1.23)**	—	—	—	**0.93, <0.001**	**0.92, <0.001**	**0.92, <0.001**	**0.87, <0.001**
**Entorhinal cortex**	**1.58 (1.23)**	—	—	—	—	**0.91, <0.001**	**0.90, <0.001**	**0.85, <0.001**
**Anterior cingulate cortex**	**1.81 (1.39)**	—	—	—	—	—	**0.93, <0.001**	**0.85, <0.001**
**Angular gyrus**	**1.86 (1.33)**	—	—	—	—	—	—	**0.87, <0.001**
**Calcarine cortex**	**1.44 (1.04)**	—	—	—	—	—	—	—

[a]Repeated Measure Analysis of Variance indicates that Aβ load in the 8 brain regions were different (F=81.2, p<0.001). A post hoc Tukey test, at 0.05 significance level, indicated that middle frontal cortex, angular gyrus, and cingulate cortex had the highest levels of Aβ load (their levels were not different), followed by superior frontal and inferior temporal cortices, then entorhinal and calcarine cortices, and finally hippocampus.
