# Supplementary material for: G-protein coupled estrogen receptor 1, amyloid-β, and tau tangles in older adults
Source: Commun Biol. 2024 May 15;7:569. doi: 10.1038/s42003-024-06272-9 (PMC11096330; doi:10.1038/s42003-024-06272-9)
Supplement: Supplementary file 5 — Reporting summary [file 42003_2024_6272_MOESM5_ESM.pdf]

nature portfolio

Corresponding author(s): Shahram Oveisgharan

Last updated by author(s): Apr 19, 2024

Reporting Summary

Nature Portfolio wishes to improve the reproducibility of the work that we publish. This form provides structure for consistency and transparency in reporting. For further information on Nature Portfolio policies, see our Editorial Policies and the Editorial Policy Checklist.

Statistics

For all statistical analyses, confirm that the following items are present in the figure legend, table legend, main text, or Methods section.

n/a	Confirmed	
☐	☒	The exact sample size (*n*) for each experimental group/condition, given as a discrete number and unit of measurement
☐	☒	A statement on whether measurements were taken from distinct samples or whether the same sample was measured repeatedly
☐	☒	The statistical test(s) used AND whether they are one- or two-sided<br>*Only common tests should be described solely by name; describe more complex techniques in the Methods section.*
☐	☒	A description of all covariates tested
☐	☒	A description of any assumptions or corrections, such as tests of normality and adjustment for multiple comparisons
☐	☒	A full description of the statistical parameters including central tendency (e.g. means) or other basic estimates (e.g. regression coefficient) AND variation (e.g. standard deviation) or associated estimates of uncertainty (e.g. confidence intervals)
☐	☒	For null hypothesis testing, the test statistic (e.g. *F*, *t*, *r*) with confidence intervals, effect sizes, degrees of freedom and *P* value noted<br>*Give P values as exact values whenever suitable.*
☒	☐	For Bayesian analysis, information on the choice of priors and Markov chain Monte Carlo settings
☒	☐	For hierarchical and complex designs, identification of the appropriate level for tests and full reporting of outcomes
☐	☒	Estimates of effect sizes (e.g. Cohen's *d*, Pearson's *r*), indicating how they were calculated

Our web collection on statistics for biologists contains articles on many of the points above.

Software and code

Policy information about availability of computer code

Data collection	*Provide a description of all commercial, open source and custom code used to collect the data in this study, specifying the version used OR state that no software was used.*
Data analysis	The analyses were done using SAS version 9.4.

For manuscripts utilizing custom algorithms or software that are central to the research but not yet described in published literature, software must be made available to editors and reviewers. We strongly encourage code deposition in a community repository (e.g. GitHub). See the Nature Portfolio guidelines for submitting code & software for further information.

Data

Policy information about availability of data

All manuscripts must include a data availability statement. This statement should provide the following information, where applicable:

- Accession codes, unique identifiers, or web links for publicly available datasets
- A description of any restrictions on data availability
- For clinical datasets or third party data, please ensure that the statement adheres to our policy

To obtain ROSMAP available resources, qualified investigators should submit an application at www.radc.rush.edu that includes study premises and a short research plan.

Human research participants

Policy information about studies involving human research participants and Sex and Gender in Research.

Reporting on sex and gender	Sex, years of education, and race data were obtained through self-report at the parental cohorts' baseline evaluation. Participants were on average 89.6 (SD=6.6) years old at the time of death, two thirds were women. Estrogen level is lower in postmenopausal women compared with men(19), which might change the interaction between GPER1 RNA expression and Aβ across sexes. In the current analyses, GPER1 RNA expression level was higher in women compared with men (13.88 (SD=0.93) vs. 13.71 (SD=0.84), p=0.001), an indirect measure of different estrogen levels in the 2 sexes. Therefore, we estimated the interactions between GPER1 RNA expression and Aβ load in the association with tau tangles in men and women separately (Figure 2). The interaction estimates were similar between women and men [(women: estimate=0.058, SE=0.039, p=0.137); (men: estimate=0.057, SE=0.054, p=0.296)]. These findings suggested that GPER1 RNA expression level modified the association between Aβ and tau tangles in men and women. Furthermore, controlling the main models for estrogen-related medications did not change the modification of the association between Aβ and tau tangles by GPER1 (Table e-1).
Population characteristics	Participants were on average 89.6 (SD=6.6) years old at the time of death, two thirds were women, 96.8% (n=1167) were non-Hispanic White, and 42% (n=507) had AD dementia. In postmortem examination, 65% (n=775) had AD pathological diagnosis.
Recruitment	Participants were from one of two longitudinal ongoing clinical pathological studies of aging and dementia, the Religious Orders Study (ROS) or the Rush Memory and Aging Project (MAP) conducted by Rush Alzheimer's Disease Center. ROS began enrollment in 1994, and eligible candidates were older nuns, priests, and brothers across the United States who were without known dementia and consented to annual clinical evaluations and brain donation at the time of death. MAP began enrollment in 1997, and eligible candidates were older Illinoisans living in retirement facilities or personal accommodations in northeastern Illinois who were without known dementia and consented for clinical evaluations and brain donations. Of 3734 ROSMAP participants with completed baseline evaluations till April 2022, 1836 had died with completed postmortem pathological assessments. Of the 1836 participants, the first 1206 whose dorsolateral prefrontal (DLPFC) tissues had been examined for transcriptomics comprised the analytic sample. RNA sequencing is ongoing.
Ethics oversight	Both studies were approved by an Institutional Review Board of Rush University Medical Center. All participants signed an informed consent and Anatomic Gift Act.

Note that full information on the approval of the study protocol must also be provided in the manuscript.

Field-specific reporting

Please select the one below that is the best fit for your research. If you are not sure, read the appropriate sections before making your selection.

☐ Life sciences ☐ Behavioural & social sciences ☐ Ecological, evolutionary & environmental sciences

For a reference copy of the document with all sections, see nature.com/documents/nr-reporting-summary-flat.pdf

Life sciences study design

All studies must disclose on these points even when the disclosure is negative.

Sample size	No sample size calculation was performed. We used all available data including ROSMAP participants who had died, had completed postmortem pathological assessments and available transcriptome data from dorsolateral prefrontal cortex tissues.
Data exclusions	We excluded participants who had died but did not undergo autopsy or did not have pathological or transcriptome data.
Replication	We replaced amyloid-beta quantified by immunohistochemistry with amyloid-beta quantified by proteomic analysis, and the findings were replicated. We replaced bulk tissue RNA expression levels with single nuclei RNA expression levels of GPER1, and the findings were replicated.
Randomization	No randomization was performed. It was an observational study, and models were controlled for age at death, sex, and education.
Blinding	Personnel who collected pathological or omics data were blind to clinical data of the participants.

Behavioural & social sciences study design

All studies must disclose on these points even when the disclosure is negative.

Study description	*Briefly describe the study type including whether data are quantitative, qualitative, or mixed-methods (e.g. qualitative cross-sectional, quantitative experimental, mixed-methods case study).*

Research sample	*State the research sample (e.g. Harvard university undergraduates, villagers in rural India) and provide relevant demographic information (e.g. age, sex) and indicate whether the sample is representative. Provide a rationale for the study sample chosen. For studies involving existing datasets, please describe the dataset and source.*
Sampling strategy	*Describe the sampling procedure (e.g. random, snowball, stratified, convenience). Describe the statistical methods that were used to predetermine sample size OR if no sample-size calculation was performed, describe how sample sizes were chosen and provide a rationale for why these sample sizes are sufficient. For qualitative data, please indicate whether data saturation was considered, and what criteria were used to decide that no further sampling was needed.*
Data collection	*Provide details about the data collection procedure, including the instruments or devices used to record the data (e.g. pen and paper, computer, eye tracker, video or audio equipment) whether anyone was present besides the participant(s) and the researcher, and whether the researcher was blind to experimental condition and/or the study hypothesis during data collection.*
Timing	*Indicate the start and stop dates of data collection. If there is a gap between collection periods, state the dates for each sample cohort.*
Data exclusions	*If no data were excluded from the analyses, state so OR if data were excluded, provide the exact number of exclusions and the rationale behind them, indicating whether exclusion criteria were pre-established.*
Non-participation	*State how many participants dropped out/declined participation and the reason(s) given OR provide response rate OR state that no participants dropped out/declined participation.*
Randomization	*If participants were not allocated into experimental groups, state so OR describe how participants were allocated to groups, and if allocation was not random, describe how covariates were controlled.*

Ecological, evolutionary & environmental sciences study design

All studies must disclose on these points even when the disclosure is negative.

Study description	*Briefly describe the study. For quantitative data include treatment factors and interactions, design structure (e.g. factorial, nested, hierarchical), nature and number of experimental units and replicates.*
Research sample	*Describe the research sample (e.g. a group of tagged Passer domesticus, all Stenocereus thurberi within Organ Pipe Cactus National Monument), and provide a rationale for the sample choice. When relevant, describe the organism taxa, source, sex, age range and any manipulations. State what population the sample is meant to represent when applicable. For studies involving existing datasets, describe the data and its source.*
Sampling strategy	*Note the sampling procedure. Describe the statistical methods that were used to predetermine sample size OR if no sample-size calculation was performed, describe how sample sizes were chosen and provide a rationale for why these sample sizes are sufficient.*
Data collection	*Describe the data collection procedure, including who recorded the data and how.*
Timing and spatial scale	*Indicate the start and stop dates of data collection, noting the frequency and periodicity of sampling and providing a rationale for these choices. If there is a gap between collection periods, state the dates for each sample cohort. Specify the spatial scale from which the data are taken*
Data exclusions	*If no data were excluded from the analyses, state so OR if data were excluded, describe the exclusions and the rationale behind them, indicating whether exclusion criteria were pre-established.*
Reproducibility	*Describe the measures taken to verify the reproducibility of experimental findings. For each experiment, note whether any attempts to repeat the experiment failed OR state that all attempts to repeat the experiment were successful.*
Randomization	*Describe how samples/organisms/participants were allocated into groups. If allocation was not random, describe how covariates were controlled. If this is not relevant to your study, explain why.*
Blinding	*Describe the extent of blinding used during data acquisition and analysis. If blinding was not possible, describe why OR explain why blinding was not relevant to your study.*

Did the study involve field work? ☐ Yes ☐ No

Field work, collection and transport

Field conditions	*Describe the study conditions for field work, providing relevant parameters (e.g. temperature, rainfall).*
Location	*State the location of the sampling or experiment, providing relevant parameters (e.g. latitude and longitude, elevation, water depth).*
Access & import/export	*Describe the efforts you have made to access habitats and to collect and import/export your samples in a responsible manner and in*

Access & import/export	*compliance with local, national and international laws, noting any permits that were obtained (give the name of the issuing authority, the date of issue, and any identifying information).*
Disturbance	*Describe any disturbance caused by the study and how it was minimized.*

Reporting for specific materials, systems and methods

We require information from authors about some types of materials, experimental systems and methods used in many studies. Here, indicate whether each material, system or method listed is relevant to your study. If you are not sure if a list item applies to your research, read the appropriate section before selecting a response.

Materials & experimental systems

n/a	Involved in the study
☐	☒ Antibodies
☒	☐ Eukaryotic cell lines
☒	☐ Palaeontology and archaeology
☒	☐ Animals and other organisms
☐	☒ Clinical data
☒	☐ Dual use research of concern

Methods

n/a	Involved in the study
☐	☒ ChIP-seq
☒	☐ Flow cytometry
☒	☐ MRI-based neuroimaging

Antibodies

Antibodies used	Antibodies specific for Aβ that were used were 6F/3D (1:50, Dako North America Inc., Carpinteria, CA); 10D5 (1:600, Elan Pharmaceuticals, San Francisco, CA); 4G8 (1:9000, Covance Labs, Madison, WI). An antibody that was specific for phosphorylated tau was AT8 (1:1000, Innogenetics, Alpharetta, GA).
Validation	Immunohistochemical analysis was performed, as previously described (ref. 6), with diaminobenzidine as the reporter and with 2.5% nickel sulfate to enhance immunostain contrast. All sections were run using identical incubation times on an automated immunohistochemical stainer in precisely timed runs. Control sections processed without the antibody, that displayed no specific staining, and positive controls were included in all runs.

Clinical data

Policy information about clinical studies

All manuscripts should comply with the ICMJE guidelines for publication of clinical research and a completed CONSORT checklist must be included with all submissions.

Clinical trial registration	*Provide the trial registration number from ClinicalTrials.gov or an equivalent agency.*
Study protocol	*Note where the full trial protocol can be accessed OR if not available, explain why.*
Data collection	*Describe the settings and locales of data collection, noting the time periods of recruitment and data collection.*
Outcomes	*Describe how you pre-defined primary and secondary outcome measures and how you assessed these measures.*

ChIP-seq

Data deposition

☐ Confirm that both raw and final processed data have been deposited in a public database such as GEO.

☐ Confirm that you have deposited or provided access to graph files (e.g. BED files) for the called peaks.

Data access links<br>*May remain private before publication.*	*For "Initial submission" or "Revised version" documents, provide reviewer access links. For your "Final submission" document, provide a link to the deposited data.*
Files in database submission	*Provide a list of all files available in the database submission.*
Genome browser session<br>(e.g. UCSC)	*Provide a link to an anonymized genome browser session for "Initial submission" and "Revised version" documents only, to enable peer review. Write "no longer applicable" for "Final submission" documents.*

Methodology

Replicates	*Describe the experimental replicates, specifying number, type and replicate agreement.*

Sequencing depth	*Describe the sequencing depth for each experiment, providing the total number of reads, uniquely mapped reads, length of reads and whether they were paired- or single-end.*
Antibodies	*Describe the antibodies used for the ChIP-seq experiments; as applicable, provide supplier name, catalog number, clone name, and lot number.*
Peak calling parameters	*Specify the command line program and parameters used for read mapping and peak calling, including the ChIP, control and index files used.*
Data quality	*Describe the methods used to ensure data quality in full detail, including how many peaks are at FDR 5% and above 5-fold enrichment.*
Software	*Describe the software used to collect and analyze the ChIP-seq data. For custom code that has been deposited into a community repository, provide accession details.*
